# Moss Fibre Gold

**Published:** 1904-01

**Authors:** B. F. Luckey


### MOSS FIBRE GOLD.

Dr. B. F. Luckey.

I should like, if possible, to bring out a discussion on the subject of moss fibre gold. I have used it practically ever since it has been on the market, and my experience has led me to confine the use of it to small approximal, buccal or palatal cavities, where there would be no stress or strain on the gold. I have not been able to so condense it as to feel that I would have any special strength in the filling. In talking about this gold with other gentlemen I have been assured that they have used it in many exposed places, in the building up of contour fillings, and to make corners on incisor teeth with good satisfaction. I have not been able to so manipulate the gold that I have felt satisfied with its strength, or felt warranted in making contour fillings of it, and it occurred to me there might be some gentleman present who has had more experience with the use of moss fibre gold than I have, and that possibly he might be able to tell us how to so manipulate the gold that we could have strong fillings in exposed places such as I have not been able to make. If it is possible to so manipulate it, it does seem to me it would be an advantage, as it is so much more easily manipulated than foils or cylinders.

Dr. Harlan: I have had quite a little experience with Watts gold, but I have not been able to manipulate it with hand pressure for contour work. When I have attempted it, while it looked very nice, it would break, but by using a fine point in an automatic mallet or an engine mallet I have been able to do much better.

Dr. Leroy: Moss fibre gold is used almost exclusively in my practice. The only deviation I have made in the last three years is to occasionally incorporate foil with it, on the surface of fillings—and it is done simply to determine whether there is any advantage in using foil in conjunction with fibre gold. As yet I am not able to say there is any advantage. I do all kinds of contour work that comes under my care with the moss fibre gold and have no hesitancy in using it in any locality where I would use foil. I build up all contours with it as readily as in cavities in the masticating surfaces of teeth.

The method of procedure is very simple. The fibre gold is supplied in sheet form and after carefully removing the cottonoid top and the cotton fibres adhering to the surface, a portion of the gold is taken from the block and placed on a mica slab and heated at first sufficiently to drive from the gold any moisture that may be present. That leaves the material fairly soft and plastic, a quality required of any gold for adaptability to tooth walls. The base of any cavity can be manipulated very easily under these conditions. When nearing the surface the gold is heated again on the mica slab until there is a change in the color of the material and the gold becomes a reddish tint; by that means the material is annealed quite thoroughly. If the operation is on the masticating surface or a contour upon an incisor tooth I heat the gold through the mica until it is quite red hot, going over the flame with each piece of gold. I use the S. S. White points made especially for the purpose; the smaller ones of the set. I rarely use the larger points. The bases of cavities are built up with them until nearly filled. In contour work I use an a Avil mechanical variable angle mallet in an engine propelled by an electric motor, which is preferable to the electric mallet. The electric mallet does not seem to work uniformly in my hands.

Dr. Fisher: Under the head of incidents of office practice, I want to call attention to a case which came to me about four months ago. The patient had been in my hands for other work some eight or nine years before, but at the time I speak of, her gums and the membranes showed every evidence of Riggs disease, although I had some doubts about its actual existence. The patient had been suffering from a severe sore throat and a physician had directed her to use as a nasal douche Siler's antiseptic tablets for her nose and throat. The effect of this wash in the mouth was most remarkable; and instead of her teeth being loose and her gums in a spongy condition, the teeth were very hard and firm and the gums perfectly healthy in a short time after using that wash. Since that time I have treated several other cases, and I believe that a good many that we treat for Riggs disease have a form of catarrh of the gums. In another case I have in mind I used borine, pyrozene, dioxygen and other preparations with no relief and effected a cure by the use of these tablets. I have used them in the case of my own sister, who had several loose teeth, with the utmost success.

